# It Takes Two: The Round-Robin Methodology for Investigative Interviewing Research

**DOI:** 10.3389/fpsyg.2018.02181

**Published:** 2018-11-13

**Authors:** Charlotte A. Hudson, Liam P. Satchell, Nicole Adams-Quackenbush

**Affiliations:** ^1^Department of Psychology, University of Portsmouth, Portsmouth, United Kingdom; ^2^School of Law and Criminology, University of West London, London, United Kingdom; ^3^Department of Criminology and Criminal Law, Maastricht University, Maastricht, Netherlands

**Keywords:** investigative interview, individual differences, dyadic research, round-robin analysis, witness, interviewer

## Abstract

Investigative interviews are complex, dyadic, and social interactions typically studied by evaluating interviewers’ questioning strategies. In field settings, interviewers naturally vary in their interviewing practice. Thus, it is important to conduct research reflective of idiosyncrasies in witnesses, interviewers, and the resulting unique pairings. This study explored sources of variation in an interview by using a “round-robin” design. Each session of the study involved five witnesses observing five separate events. Witnesses were then simultaneously, but independently interviewed by four different interviewers, or completed a self-administered written interview. This sequence was repeated until each witness had seen every event and had been interviewed by each interviewer. Over nine sessions (*N* = 45) this produced 225 total interviews. Individual interview performance (accuracy and level of detail) as well as experience (subjective ratings) were then analyzed in relation to the typical performance of the interviewer, the witness, the event, and the unique paring. We found that witnesses and interviewers could have an effect on statement quality; however, the unique interview experience variance had the greatest influence on interview performance. This study presents the round-robin methodology as a useful tool to study realistic variation in interviewer, witness, and dyad behavior. The preprint of this paper is available at psyarxiv.com/tv5gz/, and materials and data are available at osf.io/ef634/files/.

## It Takes Two to Make an Interview Go Right: Round-Robin Designs to Demonstrate Interviewer and Witness Variance

A comprehensive witness interview is paramount in solving criminal cases (Geiselman et al., 1986). The main objective of a witness interview is to acquire the most accurate, complete, and detailed account of an event (Milne and Powell, 2010). Thus, the ability to obtain comprehensive, high quality, and reliable information is crucial for interviewers in forensic settings (Hope, 2013). The present study aims to explore the influence of the interviewer, the witness and the effects of unique interview experience variance on interview quality by using a half block round-robin mock interview design.

Developing good interview protocols has been prioritized over the comprehension of interviewer–witness variation in investigative interviewing research. Those studies have improved our understanding of interview techniques; however, they lack the analytical nuances to reflect the variability of dyads, interviewers, and witnesses in practice. The design of a study influences the relevance and generalizability of the findings for applied situations. This can be significant for practitioners who are trying to implement evidence-based findings into practice (Lum et al., 2012). Thus, it is important to design studies that will further our understanding of how interviewers elicit the most reliable information while contending with aspects of the interview that are within and without their control. For example, effective communication between the witness and the interviewer is paramount for obtaining investigation relevant information (Fisher, 2010). Skilled interviewing also requires versatility (Olson et al., 2016; Christiansen et al., 2018), a sound understanding of memory processes (Hope, 2013), as well as, active listening skills, and accuracy in self-assessment (Walsh et al., 2017). However, the extent to which those interviewer traits interact with various witness traits remains under-investigated.

Witnesses reported a greater impression of being listened to when interviewers used information-gathering strategies (Vrij et al., 2006). The Enhanced Cognitive Interview (ECI; Fisher and Geiselman, 1992) is an information-gathering technique developed to maximize the amount of investigation relevant information a witness provides. The ECI is founded on evidence-based psychological principles of memory and focuses on general memory support (i.e., reinstatement of context and perspective change). The ECI also incorporates the vital role of comfort and rapport on interview procedures and includes a framework for communicating effectively with the witness (see Fisher and Geiselman, 1992). For example, interviewers are encouraged to use active listening (e.g., acknowledging when a witness responds to a question with verbalizations such as “uh huh”) as an element of building trust and understanding when employing ECI techniques (Walsh and Bull, 2012). In sum, the creators of the ECI recognized the interview as a social interaction (Melinder and Gilstrap, 2009). They created a technique that situates the interviewer in a position to direct and determine the nature and content of what the witness recalls (Randall et al., 2006).

The investigative interview with cooperative witnesses is a type of conversation. It is the responsibility of the interviewer to effectively communicate their investigative needs and facilitate the dialog in a manner that achieves the interview goals (Fisher, 2010). Within a conversation, partners work together to establish comprehension of previous utterances before initiating new contributions (Clark and Wilkes-Gibbs, 1986). In the interview conversation, the interviewer is mainly responsible for ensuring that contributions are understood. This can be exhibited as acknowledgments through repeating and paraphrasing key utterances (Clark and Wilkes-Gibbs, 1986; Vredeveldt et al., 2016). To prompt the witness for further information, interviewers may also use open-ended invitations for additional details, which are more likely to increase the accuracy of the information than focused prompting (e.g., “What happened next?” versus “Can you explain the sequence of events?”; Lamb et al., 2007). Open-ended prompts also encourage the witness to say more. However, the ability to utilize those communication skills may vary dependent on other factors present in the interview. For example, a witness may be reluctant to talk, or may not be forthcoming with the required level of detail. In those situations, the interviewer may need to employ additional prompting techniques or increase requests for clarification, and the communication skills and social flexibility of the interviewer become integral in assisting in the elicitation of further detail (Fisher, 2010).

Experimental manipulations of social skills (e.g., congeniality and rapport building) have shown that hostile interviewer behavior toward witnesses decreases the quantity and quality of reported crime-relevant details (Collins et al., 2002). An interviewer who exhibits pro-social behavior improves the ability of the witness to recall correct information, while minimizing the amount of incorrect information reported, and any susceptibility to misinformation (Vallano and Compo, 2011). Thus, interviewers displaying exaggeratedly positive or negative behavior can impact the outcome of interviews. However, rapport does not always occur in such extreme manipulations. Variability in the prosociality of individuals is well documented, and the cornerstone of many personality theories (Davis, 1980; Greene, 2000; Lee and Ashton, 2004; Essau et al., 2006; Soto and John, 2009).

Research has previously demonstrated that personality traits such as “agreeableness” relate to natural variability in cooperative tasks (Zhao and Smillie, 2015). Furthermore, anxiety traits related to social comfort are known to decrease interrogation performance in an interview context (Ono et al., 2011). Taken together, research has suggested that individual differences may affect interview quality as well as the quality of the witness statement. Successful interviewer–witness pairings may develop because of personal qualities that are made salient during interpersonal interactions (e.g., increased amiability and decreased anxiety); however, those qualities will present differentially based on the individuals involved in the interaction. Social skills, therefore, become a cumulative effect that are not easily divisible into the characteristics displayed (Tickle-Degnen and Rosenthal, 1990). Interpersonal theory suggests that an objective of interpersonal behavior is to elicit complementary responses from others (Horowitz et al., 2006). Complementary responses are seen in affiliative behavior where one part of the dyad perceives his or her partner as similar to himself or herself, and subsequently rates the interaction as more satisfactory than counterparts who considered their partner as dissimilar (Dryer and Horowitz, 1997).

Social science research prioritizes analysis more on averages than on variability in groups. The averages used in research are statistical abstractions, and result in the loss of detail in terms of individual variation. Effect sizes become distorted because of averaging and aggregating across multiple trials and participants (Brand and Bradley, 2012). Researchers of applied topics (e.g., witness interviewing) have demonstrated the effectiveness of techniques through changes in large sample averages compared to a standard interview (e.g., Fisher et al., 1989; Paulo et al., 2015). While there are benefits to demonstrating that specific interviewer techniques are, on average, more effective, applied settings are complex. Witnesses often display diverse behaviors that are not captured because it is rare for an individual to be accurately represented by aggregate research (Chamorro-Premuzic, 2016; Levine, 2016).

Individual differences are often small effects in psychological research but influence everyday life (Roberts et al., 2007). Studies of this type often require large samples to account for idiosyncratic sources of noise in data. This is problematic when attempting to efficiently conduct academic research where many witnesses and interviewers are required to fully explore interview settings. However, there are techniques that allow researchers to better account for the nature of variability that occurs in dyads (see Cronbach, 1955; Kenny and Albright, 1987) such as the “round-robin” design (Warner et al., 1979). In this paradigm, participants engage in multiple interactions with multiple partners. In a forensic context, this design is used to test how multiple interviewers engage with multiple witnesses. In the current study, we build on the existing research regarding the investigative interview with witnesses to examine the naturally varying individual differences in social skills using the round-robin methodology.

The benefit of the round-robin approach is the ability to account for the typical performance of those involved, as well as other repeated interview factors. For example, a hypothetical Interviewer A may be a better interviewer than a hypothetical Interviewer B. Thus, Interviewer A’s performance will be generally better than B’s regardless of witness interviewed. However, Interviewer A could be exposed to a witness who has poor memory, which could result in a lower quality account. Interviewer B, while a poorer interviewer on average may show more flexibility with this particular witness and the unique pairing of Interviewer B and the witness may produce a better interview. As the typical performance of the interviewer and witness becomes evident, a round-robin design allows researchers to attribute performance to individual variance and the unique factors of the event, while accounting for effects of repeated interviewing.

We expected that variation in quantity and quality of the elicited information would be due to variability in the interviewers’ and witness’ interpersonal style, and the present study is an exploratory investigation to test these assumptions. We included measures of subjective interview perception, witness personality (interviewer personalities were not measured due to the small sample size, *n* = 4), and interviewer behavior (audible acts such as prompts and verbal crutches) to measure the individual differences in interview outcomes.

## Materials and Methods

### Participants

#### Witnesses

A sample of 45 participants (22 males, 22 females, one “other”) between 19 and 38 years of age (*M* = 24.71, *SD* = 4.81), signed up as mock witnesses. Participants were recruited from a university in the United Kingdom (*n* = 20) and local community members (*n* = 25). Before taking part in the study, participants were asked to inform the researchers if they recognized any of the interviewers. Three witnesses reported meeting one of the interviewers before^1^; however, none of the witnesses or interviewers knew each other on a personal level, so the data were retained. No compensation was given for participation.

#### Interviewers

Four research assistants (two males, two females) between 19 and 29 years (*M* = 22.50, *SD* = 4.73) were trained as interviewers for all nine rounds in the study. Three interviewers had (at the time of data collection) completed 2 years of undergraduate psychology study and the fourth was a psychology postgraduate student. All interviewers were familiar with Cognitive Interview (CI) research through a series of lectures included in their university courses. The first author had 6 years of experience in interviewing in this style in a research setting at the time of data collection. A fifth “interviewer” was a self-report statement detailed in “the self-administered report” subheading below.

The four mnemonics of the CI (i.e., reinstate context, report everything, change order, and change perspective) were used to develop an example of an interview script that was provided to the interviewers. Prior to data collection, the interviewers received individual 1-h training sessions with the first author. The mnemonics of the CI were highlighted and discussed at length, and interviewers were given an opportunity to practice and receive feedback on their interviewing skills. During the training, interviewers were informed that there would be a 2-min pre-interview interaction with each witness to allow them to get acquainted with the witness. As part of the interview training sessions, the ECI framework for developing rapport was also discussed (see Fisher and Geiselman, 1992). To encourage engagement from the interviewers, their role was framed as an informal competition, with whoever averaged the most correct details as the “winner.”

### Design

This study used a half block round-robin design with nine rounds of five witnesses in each “round” (see Table 1). The same four interviewers and the written statement were constant across each round. All witnesses observed each event and were interviewed by each interviewer on one occasion. This resulted in 25 interviews being conducted per round (five witnesses participating in five interviews). Across nine rounds (a total of 45 witnesses), this led to a total of 225 interviews being conducted.

**Table 1:** The schedule for each witness per round of the design.

	Interview 1	Interview 2	Interview 3	Interview 4	Interview 5
Witness 1	Video 1	Video 2	Video 3	Video 4	Video 5
	Interviewer 1	Interviewer 2	Interviewer 3	Interviewer 4	Interviewer 5
Witness 2	Video 2	Video 3	Video 4	Video 5	Video 1
	Interviewer 3	Interviewer 4	Interviewer 5	Interviewer 1	Interviewer 2
Witness 3	Video 3	Video 4	Video 5	Video 1	Video 2
	Interviewer 5	Interviewer 1	Interviewer 2	Interviewer 3	Interviewer 4
Witness 4	Video 4	Video 5	Video 1	Video 2	Video 3
	Interviewer 2	Interviewer 3	Interviewer 4	Interviewer 5	Interviewer 1
Witness 5	Video 5	Video 1	Video 2	Video 3	Video 4
	Interviewer 4	Interviewer 5	Interviewer 1	Interviewer 2	Interviewer 3

*Interviewer 5 was a self-administered written report*.

### Materials and Measures

#### The Stimulus Videos

Five videos were sourced from the public domain ^2^ and cropped to a similar length of time (*M* = 61.20 s, *SD* = 9.96 s). Each video focused on a short confrontation involving at least two people in a public location and was rich in visual and auditory detail. Each had a unique narrative to ensure they were easily discernible from each other. One video focused on a group of skateboarding teenagers in an altercation with a security guard. One video depicted a large group of men engaged in a street fight. Another video showed two young men in a car park in an organized fight. Another video depicted a man who was attacked at a cycling event in London. The final video showed two young men arguing in an outdoor drinking establishment. The videos were presented to witnesses in a counterbalanced order. While our sample of stimuli featured only male offenders, genuine witnesses of crimes are more likely to see a male perpetrator of crime, given the sex differences in law breaking (for an overview, see Bennett et al., 2005).

#### The Interview

We developed a standardized interview script utilizing the four retrieval components of the CI. Each of these CI components was posed as an interview question to help guide the four interviewers in eliciting as much information from participants as possible. For example, the exemplary recall everything question was phrased as “I’d like you to walk me through the events as they happened, telling me every little detail you can remember. It doesn’t matter whether you think it is relevant or not, but please don’t guess. Think about the events of the video, who was involved, where it happened. Whenever you are ready, I’d like for you to tell me everything that happened in the video.” We instructed each interviewer to ask the questions using their own words in any order they wished. This allowed for idiolect and questioning preference, which is known to naturally vary in studies of CI trained police officers (see Dando et al., 2008). A copy of the example script is available at osf.io/dq2ag/.

#### The Self-Administered Report

Witnesses also reported the details of one witnessed event on a computer. This step was taken to observe the effect of an interview without social interaction. To follow cognitive interview structure as closely as possible in the written format, we asked witnesses to complete a written free recall, inspired by the Self-Administered Interview (SAI, Gabbert et al., 2009) which does not require an interviewer to be present. While we did not utilize the SAI, we drew upon the same premise of employing a written free recall tool that does not involve a dyadic interaction. The SAI was developed as a generic written-response recall tool that could be used to gather high-quality information from witnesses shortly after a variety of events (Gabbert et al., 2009). The SAI takes the form of a questionnaire booklet, that draws on memory theory and empirical research to elicit comprehensive free recall witness statements in their own words. Our written recall tool differed in that it was less thorough than the SAI, and simply acted as a written recall tool. Our tool also contained the same questions from the CI training script given to interviewers. A copy of the written interview script is available at osf.io/9px72/.

#### Subjective Experience Measures

To capture the subjective experience of being in the interview (and capture non-observational details, such as internal state of mind), all witnesses and interviewers completed rating scales immediately after each interview. The witness subjective rating scales are available at osf.io/2zfrm/, and interviewer subjective rating scales are available at osf.io/byxp6/. Perceptions were rated on a scale of 0% (*strongly disagree*) to 100% (*strongly agree*). Witnesses and interviewers rated their perception of the witness’ memory of the crime video stimulus. They were asked the extent to which they agreed that the witness: (i) was able to remember everything in the video, (ii) understand the event, (iii) give an accurate account, (iv) did not include omissions in their account, and (v) did not include commissions in their account. The five ratings were evaluated in a highly similar manner by the witnesses (ICC = 0.90 95% CI [0.88, 0.92], *p* < 0.001) and the interviewers (ICC = 0.90, 95% CI [0.88, 0.92], *p* < 0.001). Therefore, the response to “remember everything” in the interview (as a holistic judgment by witnesses themselves and by the interviewers) was retained as a representative variable for subjective evaluations of witness memory (see Table 2).

**Table 2:** Descriptive statistics of percentage endorsement of witness memory, witness interview skill and interviewer score.

Subjective rating	Rater	Mean	*SD*	Min	Max	Skew
Witness memory	Witness	69.29	19.84	10.00	100.00	-0.89
	Interviewer	73.87	16.97	10.00	100.00	-0.90
Good witness	Witness	75.72	19.57	0.00	100.00	-1.16
	Interviewer	84.64	15.26	10.00	100.00	-2.09
Good interviewer	Witness	79.06	21.61	0.00	100.00	-1.25
	Interviewer	78.62	16.68	30.00	100.00	-0.59

Interviewers and witnesses rated their perception of the interviewers’ performance and reported on the extent to which the interviewer: (i) was competent, (ii) asked clear questions, (iii) seemed nice, (iv) was confident, and (v) overall, was a “good interviewer.” The interviewers were highly consistent in their self-evaluation (ICC = 0.96, 95% CI [0.95, 0.97], *p* < 0.001) and the witnesses less so (ICC = 0.63, 95% CI [0.55, 0.70], *p* < 0.001) in their evaluation of the interviewers. The reliability was still good, so for efficiency we retain the “good interviewer” question as a representative of positive interviewer presence (see Table 2). Finally, witnesses and interviewers rated the witness interview performance. They rated the extent to which the witness: (i) gave a credible account, (ii) gave a clear account, (iii) was a nice person, (iv) was confident, and (v) overall, was a “good witness” in the interview. As with the above, ratings to these questions were highly consistent for witnesses (ICC = 0.91, 95% CI [0.89, 0.93], *p* < 0.001) and interviewers (ICC = 0.96, 95% CI [0.95, 0.97], *p* < 0.001). The good witness question is retained as a representative variable of positive witness interview presence (see Table 2).

For interpretive caution, we note that all participants (interviewers and witnesses) were generally complimentary of each other, which was demonstrated in the interviewers’ reports that witness memory was significantly better than the witnesses’ self-evaluation [*t*(179) = 2.50, *p* = 0.013, *d* = 0.37]. The pattern of higher ratings for the other person in the interview was the same for rating a good witness [*t*(179) = 4.46, *p* < 0.001, *d* = 0.67], and a good interviewer, [*t*(179) = 2.44, *p* < 0.001, *d* = 0.37], with small effects for two out of the three cases.

#### HEXACO Personality Assessment

We attempted to account for witness individual differences using an existing personality framework (HEXACO-60 personality inventory; Ashton and Lee, 2009). The HEXACO-60 is a well-established tool consisting of 60 questions designed to measure: Honesty-Humility (H), Emotionality (E), Extraversion (X), Agreeableness (A), Conscientiousness (C), and Openness to Experience (O). These traits have been implicated in cooperation (Zhao and Smillie, 2015) and witness research (Ono et al., 2011). It is important to note that quantifying individual differences generally requires large sample sizes (for more on relational analyses, see Schönbrodt and Perugini, 2013). With the sample size in the present study it is possible that any notable correlations would be the result of Type I error. In fact, we found no relationships between personality and sample outcomes in our data. This may also be the result of Type II error, and there may be undetected effects for the wider population as well. With those concerns in mind, we chose to report the collection of the personality data (for transparency), but decided to present the personality findings in the Supplementary Materials to avoid undue attention on potentially spurious findings.

#### Interview Coding

Each interview was transcribed, and coded for correct, incorrect, and confabulated details. The correct details were further quantified as coarse grain detail or fine grain detail (Ackerman and Goldsmith, 2008). Each transcript was first coded for number of details provided (e.g., “A young guy, wearing a white, checkered shirt was shouting,” would contain five details; the descriptors “young,” “guy,” “white,” and “checkered shirt” and the action “shouting”). These details were checked against the video footage to be classified as correct (i.e., accurate description of the video), incorrect (i.e., inaccurate description of the video), or confabulated (i.e., did not occur in the video). Each correct detail was further classified as either fine grain (e.g., “white, checkered shirt” would be two fine grain details) or as coarse grain (e.g., “young”). A second coder evaluated a subset (*n* = 22) of transcripts, and the disagreements in coding were discussed by the two coders at length to a point of resolution. There was good inter-rater reliability between the two coders on the number of Correct (ICC = 0.95, 95% CI [0.89, 0.98], *p* < 0.001), Incorrect (ICC = 0.94, 95% CI [0.84, 0.97], *p* < 0.001), Confabulated (ICC = 0.89, 95% CI [0.73, 0.95], *p* < 0.001), Fine grain (ICC = 0.93, 95% CI [0.83, 0.97], *p* < 0.001), and Coarse grain (ICC = 0.89, 95% CI [0.74, 0.96], *p* < 0.001) details in the statements.

#### Behavioral Coding

The audio recordings were also coded to determine if the interviewer’s verbal behavior could quantify interviewer variation. Two independent raters coded the audio recordings for: congenial signaling, laughter (appropriate, inappropriate, and non-humorous), pauses, speech disturbances (verbal crutches and facilitation), focus (veering away from or back to the topic), questioning type (leading, suggestive, asking for clarification, and requesting extra information), prompts, interruptions, talking over the witness, and offering information to the witness. Those verbal behaviors were selected to examine how the interviewers presented themselves within the interaction.

Some behaviors were infrequent in the interviews, and therefore, were not suitable for meaningful statistical analysis. The specific behaviors that were not present included: interviewer deviation from the topic (not present in 80.60% of cases), talking over the witness (87.60%), interrupting the witness (81.20%), offering information (94.70%), going off-topic (97.10%), returning on topic (98.80%), pausing (98.80%), non-humorous laughing (92.90%), and inappropriate laughing (84.70%). The further categories reported in Table 2 were more present, but still skewed toward absence. It is most appropriate to reduce such data to binary values for analysis (“this behavior occurred” or “this behavior did not occur”) to avoid influencing scale-based analysis techniques with skewed data. Table 3 reports on the raw frequencies of the coded interviewer behaviors and the descriptive statistics of the data when presented as binary values. These nine behaviors were retained for analysis as binary variables.

**Table 3:** The raw data frequency and percentage present of binary coding of the interviewer behaviors.

	Frequency of occurrences			Binary coding	Inter-rater reliability
Interviewer behavior	Mean	Minimum	Maximum	% Present	κ/α
Facilitation	16.56	0.00	97.00	78.20	0.82/0.91
Crutches	2.99	0.00	19.00	60.00	^a^
Congenial	1.52	0.00	9.00	56.50	0.67/0.81
Suggestive	0.78	0.00	9.00	28.20	1.00/1.00
Leading	1.06	0.00	16.00	21.80	1.00/1.00
Clarify	0.67	0.00	13.00	24.70	0.92/0.96
Laugh-humor	1.85	0.00	26.00	49.40	0.80/0.90
Prompt	1.90	0.00	19.00	26.50	1.00/1.00
Extra	0.34	0.00	6.00	24.70	^a^

^a^*The reliability statistics for Crutches and Extra are not calculable due to a lack of variance in the variables in the matched sample. In 37 cases, there was strong agreement (Crutches = 89% agreement and Extra = 100% agreement) between two coders that the behaviors were not present*.

A subset of (*n* = 37) interviews were second coded for inter-rater reliability. With binary variables, it is most appropriate to use Cohen’s κ to assess agreement; however in Table 3, we also report Cronbach’s α for readers unfamiliar with κ. Both measures show similar results, with good agreement between the coders.

### Procedure

On arrival, the five witness participants were given details about the study as a group and were also given an opportunity to ask questions before individually reading and signing informed consent. Witnesses then independently completed a demographic questionnaire, and the HEXACO-60 personality inventory.

Witnesses were then taken to separate research cubicles where they watched one of five short real crime videos. After the video finished, there was a 3-min delay while each witness moved to a new research cubicle. Witnesses were moved to new cubicles to avoid context-based cues from the room in which they witnessed the stimulus video. Four of the witnesses were met by the four interviewers and given 2 min to get acquainted. That section of the interaction was not scripted, or audio recorded. The witness who completed the scripted questions on the computer was invited to work on an unrelated word search task for 2 min to mimic the delay experienced by the other participants during the social period prior to the interview. After 2 min, the interviewers began the interview, and the witness who self-administered the interview was prompted to start typing their responses on the computer.

The interviewers used the exemplary script to develop their own versions of the four key ECI-based questions. The questions were framed in the interviewers’ own words, and they further had the opportunity to follow up with questions they thought would help gather additional information. Each interviewer was informed that they had 8 min to complete the interview, and were provided with a “2 min remaining” notification at 6 min (that time frame was established during previous pilot tests). On average, interviews lasted approximately 7 min 31 s (*SD* = 29.19 s). Likewise, the witness who interacted with the computer was verbally given a 2-min warning to complete his or her report.

Once the interviews were completed, the interviewers left the room and the researcher returned. At this point, both the interviewers and witnesses completed the three-part subjective ratings of the interview. Once the ratings were completed, the witnesses moved to a different research cubicle, watched a different crime video, and the process repeated over four more rounds. The presentation of real crime videos was fully counterbalanced so that no witness saw the same video twice, no interviewer asked questions about the same video twice within one round, no witness met the same interviewer twice, and no witness was interviewed in the same room twice. Once all the rounds were completed, witnesses were thanked for their time, they were provided the opportunity to ask any questions, and they were fully debriefed. Data collection took place over a period of 3 days.

### Data Analysis

We accounted for the influence of the witness’ typical performance, the interviewer’s typical interview, the effect of experiencing previous interviews, and the effect of the crime event witnessed on the coded features of the interview by using a linear mixed model (using the R package lme4; Bates et al., 2015). Repeated linear mixed models are well suited for analyzing round robin data as they enable the analysis of nested data, by plotting differing regression intercepts for each interviewer, witness, event, and time point. A standardized ratio of [coded] details to total details is plotted against a constant (*y* = 1) and variance in the model was accounted for using the interview features as random effects (the witness, the interview, the number of preceding interviews, and the observed event). Standardized scores for the detail ratio used for the mixed model analysis were included in the Supplementary Materials. Variation not accounted for in this model, termed “residual” variance, was the influence of non-experimental features generated by the unique qualities of that particular interview. That included the pairing of the witness to the interviewer, as well as other potential unique events in the room, which we termed “unique interview experience variance.” We used those models to attribute variance from the nested aspects of the data (the interviewers, witnesses, events, and time points) in witness performance and interviewer behavior.

Partial correlations, controlling for the aforementioned nested nature of the data, were used to evaluate the relationship between the binary variables on interviewer behavior and the witness interview performance. Those function as point biserial correlations, using a binary correlate.

Partial correlations, again accounting for the nested nature of the data, were also used to test the relationship between the subjective evaluations of the interview (perceived witness memory, witness competency, and interviewer skill) and the interview outcomes.

## Results

### Factors Accounting for Interview Performance

Table 4 presents the influence of the interview features on coded details. It is important to note that the observed event and number of preceding interviews are negligible predictors of coded details. Those findings suggested that despite the expected practice effects, and the chance certain crime videos were more memorable than others, order effects and stimuli choice did not influence the variables of interest.

**Table 4:** The standardized estimates (SD) of witness, interviewer, practice, witnessed event, and residual variance influence on the interview performance.

	Interview features				
Coding category^1^	Witness	Interviewer	Practice^2^	Event^3^	Residual
Correct details	0.25 (0.50)	0.27 (0.52)	0.00 (0.05)	0.00 (0.01)	0.52 (0.72)
Correct fine grain details	0.22 (0.47)	0.19 (0.43)	0.00 (0.00)	0.02 (0.13)	0.62 (0.79)
Correct coarse grain details	0.11 (0.33)	0.18 (0.42)	0.00 (0.06)	0.00 (0.00)	0.76 (0.87)
Incorrect details	0.01 (0.11)	0.06 (0.25)	0.00 (0.00)	0.04 (0.20)	0.91 (0.96)
Confabulated details	0.21 (0.46)	0.15 (0.38)	0.03 (0.17)	0.00 (0.05)	0.66 (0.81)

^1^*All variables are standardized values of the ratio of the detail category out of total number of details. ^*2*^The practice effect over time, that is the number of previous events witnessed and interviews participated in. ^*3*^The event witnessed by participants*.

**FIGURE 1:**
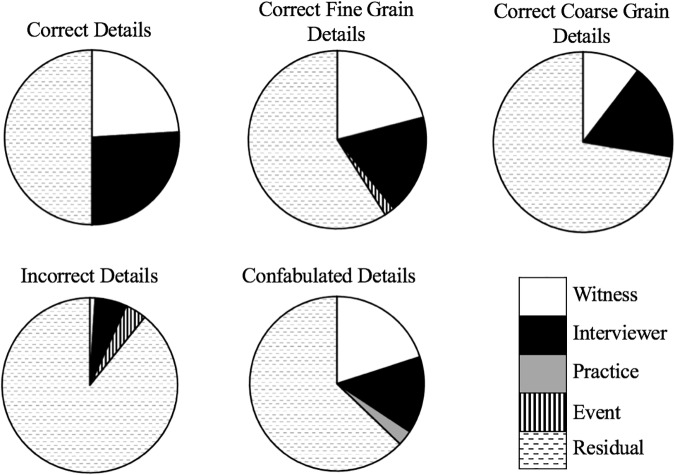
Proportional representation of the variance explained in the linear mixed model presented in Table 3.

In a hypothetical perfect interview, all variance in the ratio of correct details to total details would be entirely predicted by the quality of the witnesses’ memory, whereby the better the quality of the witness’ memory, the greater number of correct details they report in the interview. Here, we found that the average attribution of variance to the witness, across coding categories, was 0.16. The influence of the witness on correct, confabulated, and fine grain details was higher (see Figure 1). That suggested witness memory was more influential on those features than incorrect and coarse grain details. The interviewers’ style and influence on the interview did not have as strong an effect on the undesirable incorrect or confabulated details. However, we found evidence that an effective interviewer can increase the ratio of correct details and fine grain details (Table 4). The greatest influence on witness performance was the unique interview experience variance (the residual dyad effects). That effect was for all coded memory criteria (see Figure 1).

### Interviewer Behavior and Witness Performance

Table 5 presents the relationship between interviewer behavior and witness interview performance. As before, many features did not correlate; however, three effects were of noteworthy size (Ferguson, 2009) and one was significant when considering a conservative alpha threshold of *p* < 0.001. The presence of congenial interviewer behavior negatively correlated with the ratio of correct details [*r*_p_(164) = -0.22, *p* = 0.004]. Furthermore, congenial interviewer behavior [*r*_p_(164) = -0.29, *p* < 0.001] and requests for additional information [*r*_p_(164) = -0.22, *p* = 0.005] negatively correlated with coarse grain details.

**Table 5:** Partial point biserial correlations between coded details and presence of interviewer behavior (controlling for witness, interviewer, practice effects, and event witnessed).

	Ratio of coded detail type				
Interviewer behavior	Correct	Incorrect	Confabulated	Fine grain	Coarse grain
Facilitation	0.18	-0.03	0.14	0.07	0.16
Crutches	0.09	-0.07	-0.03	0.02	0.09
Congenial	-0.22*	0.06	-0.13	-0.03	-0.29**
Suggestive	0.07	-0.17	0.07	-0.02	0.18
Leading	0.03	-0.04	0.12	-0.11	0.15
Clarify	-0.11	0.02	0.00	-0.09	-0.06
Laugh-Humor	0.07	-0.08	0.04	0.01	0.09
Prompt	-0.03	-0.07	0.16	-0.15	0.13
Extra	-0.15	0.04	-0.05	-0.01	-0.21*

^∗^*p ≤ 0.005, ^∗∗^*p* < 0.001*.

Table 6 presents the repeated linear mixed model for the interviewer behaviors. What is notable in the table is that the likelihood for an interviewer to engage in facilitation, prompting the witness, and suggestive or leading questioning was largely explained by interviewer variance. Crutches, congenial behavior, clarifications, humorous laughter, and asking for extra details showed strong influences by both the interviewer and the uniqueness of the event.

**Table 6:** The standardized estimates (SD) of witness, interviewer, practice, witnessed event, and residual variance influence on the interview behavior.

	Interview features				
Interviewer behavior	Witness	Interviewer	Practice^1^	Event^2^	Residual
Facilitation	0.04 (0.19)	1.25 (1.12)	0.00 (0.03)	0.01 (0.08)	0.16 (0.40)
Crutches	0.15 (0.39)	0.42 (0.65)	0.00 (0.00)	0.00 (0.00)	0.58 (0.76)
Congenial	0.00 (0.02)	0.32 (0.56)	0.00 (0.00)	0.01 (0.07)	0.79 (0.89)
Suggestive	0.00 (0.00)	0.66 (0.81)	0.00 (0.00)	0.00 (0.04)	0.50 (0.71)
Leading	0.03 (0.19)	0.56 (0.75)	0.00 (0.00)	0.02 (0.14)	0.53 (0.73)
Clarify	0.06 (0.25)	0.34 (0.59)	0.00 (0.00)	0.00 (0.00)	0.69 (0.83)
Laugh-humor	0.07 (0.27)	0.38 (0.62)	0.01 (0.12)	0.00 (0.00)	0.67 (0.82)
Prompt	0.04 (0.19)	0.85 (0.92)	0.00 (0.00)	0.00 (0.03)	0.29 (0.54)
Extra	0.18 (0.43)	0.23 (0.48)	0.02 (0.13)	0.00 (0.00)	0.65 (0.80)

^1^*The practice effect over time, that is the number of previous events witnessed and interviews participated in ^*2*^The event witnessed by participants*.

### Subjective Evaluation and Interview Performance

There was only limited evidence that subjective evaluations of the interview were related to coded outcomes (Table 7). All correlations were of a notably small size. No correlation met the conservative *p* < 0.001 estimate of significance (due to running 30 tests at a *p* < 0.05 threshold), with the most notable correlation being between interviewer perceptions of witness memory and the ratio of correct details, *r*_p_(164) = 0.23, *p* = 0.003. Overall, there was no convincing evidence of participants’ awareness of their performance or their partners’ performance.

**Table 7:** Partial correlations between subjective evaluations and coded interview details controlling for witness, interviewer, practice effects, and event witnessed.

	Witness memory		Good witness		Good interviewer	
Coding category	W-rated	I-rated	W-rated	I-rated	W-rated	I-rated
Correct details	0.17	0.23	0.10	0.17	-0.04	0.18
Correct fine grain details	0.08	0.16	0.14	0.15	0.01	0.07
Correct coarse grain details	0.18	0.14	0.03	0.09	-0.04	0.20
Incorrect details	0.06	0.08	0.04	0.11	-0.05	-0.01
Confabulated details	0.09	0.09	-0.12	-0.05	-0.02	0.08

*W-rated, witness-rated; I-rated, interviewer-rated*.

## Discussion

In this study, we presented the round-robin methodology as a promising tool for interviewing research. We demonstrated how the amount of correct fine grain, correct coarse grain, incorrect, and confabulated detail reported in an interview can be attributed largely to the unique interview experience, more than solely to interviewer questioning strategy or witness memory. We found no evidence of confounds due to repeated measures (practice or fatigue), or stimulus (crime video) on interview performance. Moreover, we did not find evidence that our particular measures of subjective experience, personality, or many of the interviewer behaviors greatly affected interview performance in this study.

Interviewer behavior provided limited evidence to explain overall interview performance, as interviewer congeniality related only to decreased correct and coarse grain details. This was a surprising finding, which is contrary to evidence found in both conversation (Clark and Wilkes-Gibbs, 1986) and interpersonal theory (Horowitz et al., 2006). We suggest that it is possible the witnesses may have perceived interviewer congeniality as praise, and therefore, the witnesses believed they had provided sufficient information. This could have then resulted in decreased recall effort and consequently a decrease in interviewer effectiveness. Further research is needed to fully explore that finding and draw more definitive conclusions.

There was evidence that these interviewer behaviors were also generated as product of unique interview experiences. That finding highlights the benefits of using round-robin designs to investigate variance effects on interview effectiveness. The repeated rounds offer a profile of a participant’s typical performance across contexts (interviews, events, etc.). With this understanding of typical performance, deviations from the interviewer or witness’ norm behavior can be analyzed. It is reasonable to ask (of any memory experiment) if the repeated interview process affected the results. The mixed model analysis conducted here allowed us to demonstrate the extent any such repeated measures artefacts had on results. We found negligible amounts of variance resulting from practice effects or unique qualities to the stimulus videos. That finding suggests that the round-robin design is a good methodology to adopt when examining the dyadic qualities of investigator-witness type interactions. It is a limitation of the current study that we could not establish what proportion of the residual variance was a result of dyadic pairings, and what proportion was a result of other noise within the data. However, by coding for additional features such as non-verbal behavior, future round robin studies could further explore the unique interview experience variance that contributes to successful dyadic pairings and may help determine which factors influence interviewer behavior.

While many aspects of the interview performance and interviewer behavior were attributable to the unique interview experience (such as verbal crutches, asking for clarification, and humorous laughter), some of the more problematic behaviors were primarily generated by the interviewers. For example, suggestive and leading questioning, which are considered inappropriate practice (Oxburgh et al., 2010), as well as engaging in facilitation and prompting the witness were mostly accounted for by the interviewer. Therefore, there are opportunities to specifically train interviewers to avoid any problematic behavior (not “created” in interaction with witness).

There was evidence of inaccurate self and other appraisal ratings for interview performance, suggesting a positivity bias (Sears, 1983). Both witnesses and interviewers were complimentary of each other’s performance, with the data showing a pattern of high ratings given across the board (average ratings of performance ranged from 69 to 85%). There was limited evidence that subjective evaluations of the interview were related to meaningful interview outcomes. Those which did relate, correlated with correct but not incorrect or confabulated details. We speculate that this general lack of relationship between subjective and objective interview features could be a consequence of a lack of critical analysis by both witnesses and interviewers – for own- and partner performances.

While the 8-min time limit imposed on our interviews did not reflect real life forensic interviews, the time limit was set because all participants needed to interact with partners on a set schedule for practicality purposes. Thus, a high level of control was required for the round robin design. Our study also deviated from police–witness interviews in that an opportunity to build rapport was structured into the interviews to encourage affiliative behavior. That paired with the low-stress and collegial environment could have created increased positivity toward all partners (Horowitz et al., 2006), which may not be present in applied settings.

The ECI is a well-designed strategy to elicit information in a collaborative, prosocial manner (Clarke et al., 2011; Fisher and Geiselman, 1992; Tickle-Degnen and Rosenthal, 1990). Invariance in participant subjective approval could be evidence of the effectiveness of the technique putting witnesses at ease. However, this explanation fails to account for the high ratings given by the interviewers. Our interviewers were not practitioners and sample-wide high self-rating suggest that they could use training on their critical self-evaluation. Self-reflection is an important part of the PEACE framework that has been used in the United Kingdom since 1992 (Clarke et al., 2011) and is designed to help practitioners improve their own interviewing ability and avoid skill fade. Using the framework encourages interviewers to consider their conduct during the interview (e.g., was there any use of inappropriate questions, what the consequences may be, how could they have re-phrased the question), and what their appropriate next steps should be. Future research that employs round-robin methodology with interviewing professionals (as opposed to mock interviewers) could elicit key insights into self-evaluation competency. That is important as most police officers report being dissatisfied with the PEACE model investigative interview training (Dando et al., 2008). Demonstrating the importance of self-evaluation in this way could help interaction with practice.

By using repeated testing to better understand participant variation, round-robin methodologies are powerful and efficient designs. This allows small scale studies to provide a strong statistical understanding of the data. We used the round robin approach to explore the effect of individual differences on performance in an interview setting. More generally, our study demonstrated the possibility of this methodology for interviewing research and the analytical potential of larger studies following the procedure we outlined in this paper. Future research in this area could look to identify specific personality traits in witnesses and interviewers that are beneficial (or detrimental) to their interactions, and then seek a way to emphasize (or curtail) these features.^3^ For example, agreeableness has been linked to greater levels of co-operation from a partner (Zhao and Smillie, 2015), and therefore it is possible a saliently agreeable interviewer could encourage a witness to say more than a non-agreeable interviewer.

There were intriguing early signs of dyad-generated interviewer behavior on interview quality. Further research could explore approaches to coding and quantifying individual differences in temperament, behavior, and reciprocity. For example, imitation (mirroring or synchrony) is a fundamental aspect of human interaction, instrumental to interpersonal bonding and social cohesion (Brambilla et al., 2016; Semin, 2007; Semin and Cacioppo, 2008). We did not video record our interviews; however, coding for mirroring behavior could help explain the power of dyadic residual factors on interviewing performance. Moreover, other personality measures that are more oriented toward interpersonal sensitivity such as empathy (Davis, 1980) or callous–unemotional disposition (Essau et al., 2006) may be effective for explaining the variance in dyadic engagement. Further expanding the number of interviewers used between rounds (*K* > 5) would also allow a better understanding of interviewer variance.

In summary, we advocate replication and wider use of this methodology. Conducting interviewing research for applied domains requires an understanding that there is variance in the way each interviewer will engage with each witness. Interviewing research benefits from the words of the leading personality psychologist (Eysenck, 1965, p. 8); “Individuals do differ…and it seems to me that psychology will never advance very far without a recognition of the complexities which are produced by this fact.”

## Data Availability

Data were collected by CH and research assistants (noted in Section “Acknowledgments”) between 29 March 2017 and 31 March 2017. The datasets analyzed for this study, entitled “Round Robin (1) .csv” and “StandardisedInterviewRR.csv,” can (respectively) be found on the Open Science Framework at: https://mfr.osf.io/render?url=https://osf.io/qkcg4/?action=download%26mode=render

https://mfr.osf.io/render?url=https://osf.io/mr4a9/?action=download%26mode=render.

The dataset key for this study, entitled “roundrobinstudy_data set_key.txt” can be found on the Open Science Framework at: https://mfr.osf.io/render?url=https://osf.io/vnc2f/?action=download%26mode=render.

## Ethics Statement

This study was carried out in accordance with the recommendations of both British Psychological Society and American Psychological Association guidelines, University of Portsmouth Science Faculty Ethics Committee, with written informed consent from all subjects. All subjects gave written informed consent in accordance with the Declaration of Helsinki. The protocol was approved by the University of Portsmouth Science Faculty Ethics Committee.

## Author Contributions

CH and LS designed and developed materials. LS conceived the study. CH collected data. CH and NA-Q coded written and behavioral data. CH and NA-Q contributed to selection of coding criteria. LS conducted data analysis. CH prepared the manuscript with comments and edits from LS and NA-Q.

## Conflict of Interest Statement

The authors declare that the research was conducted in the absence of any commercial or financial relationships that could be construed as a potential conflict of interest.

## Footnotes

1 Three of the witnesses had previously met the same interviewer while participating in an unrelated research study. The witnesses described the nature of their interaction as neutral and professional.

2 YouTube.com

3 We attempted to quantify the differing qualities of the witnesses through the use of personality measures (HEXACO). However, personality assessment measures typically require more variation within a sample to detect effects (traditional pair-wise relational analyses do not stabilize until N > 250; Schönbrodt and Perugini, 2013), and thus with our sample size, we lacked the power to attribute performance to witness personality (at N = 45) and interviewer behavior (at K = 4).
