# Supplementary material for: It Takes Two: The Round-Robin Methodology for Investigative Interviewing Research
Source: Front Psychol. 2018 Nov 13;9:2181. doi: 10.3389/fpsyg.2018.02181 (PMC6243032; doi:10.3389/fpsyg.2018.02181)
Supplement: Supplementary file 1 [file Data_Sheet_1.pdf]

Supplementary Material

It Takes Two: The Round-Robin Methodology for Investigative Interviewing Research

Charlotte A. Hudson*, Liam P. Satchell, Nicole Adams-Quackenbush

*** Correspondence:** Charlotte Hudson: charlotte.hudson@port.ac.uk

1 Supplementary Data

We used the HEXACO-60 measure of personality to quantify witness individual differences and relate this to interview performance. This well-established tool consists of 60 questions measuring Honesty-Humility (H, in our sample M_H= 3.17, *SD*= .56), Emotionality (E, M_E= 2.91, *SD*= .63), Extraversion (X, M_X=3.78, *SD*= .81), Agreeableness (A, M_A= 3.09, *SD*= .54), Conscientiousness (C, M_C= 3.80, *SD*= .84), and Openness to Experience (O, M_O= 3.83, *SD*= .49). Partial correlations (controlling for witness, interviewer, practice effects and event witnessed) were conducted between the HEXACO personality traits to the coded details (see Table S1). We found no overall effects relating witness personality to interview performance. The strongest correlation was between witness extraversion and the ratio of incorrect details reported, $r_p(200) = .14$, $p = .047$, but this correlation was small and does not meet our conservative criteria for corrected significance.

2 Supplementary Tables

Table S1.

Partial correlations between witness personality and coded interview details controlling for witness, interviewer, practice effects and event witnessed.

	Witness Personality Trait					
Coding Category	H	E	X	A	C	O
Correct Details	-.06	-.09	-.04	-.05	.04	-.10
Incorrect Details	.01	-.07	.14	.03	-.09	.02
Confabulated Details	-.06	.08	.10	-.07	-.12	-.02
Fine Grain Details	-.08	-.11	-.04	-.05	.06	-.04
Coarse Grain Details	-.02	.01	-.05	-.07	.03	-.12

Notes. H = Honesty-Humility, E = Emotional Stability, X = Extraversion, A = Agreeableness, C = Conscientiousness, O = Openness.

Table S2.

Standardised scores (SD) of the detail ratios used for the mixed model analysis

	Correct	Correct Fine Grain Details	Correct Coarse Grain Details	Incorrect	Confabulated
Interviewer 1	0.04 *(0.91)*	0.05 *(0.85)*	0.04 *(1.04)*	0.00 *(0.83)*	0.36 *(1.03)*
Interviewer 2	-0.30 *(0.60)*	0.09 *(0.81)*	-0.57 *(0.64)*	0.27 *(1.16)*	-0.18 *(0.54)*
Interviewer 3	0.31 *(1.04)*	0.29 *(1.16)*	0.15 *(0.90)*	0.25 *(1.23)*	0.07 *(0.68)*
Interviewer 4	0.63 *(1.03)*	0.35 *(1.09)*	0.61 *(1.13)*	-0.03 *(0.98)*	0.33 *(1.50)*
Interviewer 5	-0.76 *(0.62)*	-0.76 *(0.48)*	-0.28 *(0.76)*	-0.43 *(0.51)*	-0.63 *(0.34)*

Notes. Interviewer 5 was a self-administered written report.
